# Comparative cardiovascular outcomes of renin–angiotensin system inhibitors in patients receiving maintenance hemodialysis: a large real-world cohort study

**DOI:** 10.3389/fphar.2026.1833065

**Published:** 2026-06-15

**Authors:** Jheng-Yan Wu, Keng-Wei Lee, Sheng-Chi Huang, Hsuan-Yuan Chang, Yu-Min Lin

**Affiliations:** 1 Department of Nutrition, Chi Mei Medical Center, Tainan, Taiwan; 2 Department of Public Health, College of Medicine, National Cheng Kung University, Tainan, Taiwan; 3 Division of Cardiology, Department of Internal Medicine, Chi Mei Medical Center, Chiali, Tainan, Taiwan; 4 Department of Medical Education, Chi Mei Medical Center, Tainan, Taiwan; 5 Division of Hepatogastroenterology, Department of Internal Medicine, Chi Mei Medical Centre, Tainan, Taiwan; 6 Division of Cardiology, Department of Internal Medicine, Chi Mei Medical Center, Tainan, Taiwan

**Keywords:** angiotensin receptor blockers, angiotensin-converting enzyme inhibitors, cardiovascular outcomes, end-stage kidney disease, hemodialysis, real-world evidence, renin–angiotensin system inhibitors

## Abstract

**Background:**

The comparative cardiovascular effectiveness of different renin–angiotensin system (RAS) inhibitors in patients receiving maintenance hemodialysis remains uncertain, and current guideline recommendations largely assume therapeutic equivalence between angiotensin-converting enzyme inhibitors (ACEI) and angiotensin receptor blockers (ARB).

**Methods:**

Using the TriNetX multi-institutional database, we identified adults with ESKD who newly initiated ARB or ACEI therapy from 2006 to 2025. An active-comparator new-user design and 1:1 propensity score matching were applied. The primary outcome was 1-year major adverse cardiovascular events (MACE: myocardial infarction [MI], stroke, or all-cause mortality). Secondary outcomes included individual MACE components and hyperkalemia. Hazard ratios (HRs) were estimated using Cox models, and negative-control analyses assessed residual confounding.

**Results:**

After matching, 55,894 patients were included. ARB was associated with a lower risk of MACE compared with ACEI users (30.3% vs. 35.0%; HR 0.85; 95% CI 0.83–0.88). Stroke (HR 0.90; 95% CI 0.86–0.94) and all-cause mortality (HR 0.75; 95% CI 0.71–0.78) were also associated with a lower risk, while MI risk was similar (HR 0.99; 95% CI 0.94–1.03). Hyperkalemia rates were comparable. Subgroup findings consistently favored ARBs across age, sex, diabetes, heart failure, CAD, and PAD strata. Negative-control outcomes showed no significant associations.

**Conclusion:**

In this large real-world cohort of patients receiving maintenance hemodialysis, initiation of ARB was associated with lower risks of major adverse cardiovascular events, stroke, and all-cause mortality compared with ACEI. These findings suggest a potential difference in observed cardiovascular outcomes between ARBs and ACEIs in the hemodialysis population; however, causal inference is limited by the observational design.

## Introduction

End-stage kidney disease (ESKD) remains a major public health challenge in the United States. According to the 2023 United States Renal Data System (USRDS) report, the ESKD receiving maintenance hemodialysis population continues to grow, with persistently high mortality rates despite recent advances in kidney care (Johansen et al., 2024). Diabetes remains the leading cause of kidney failure, followed by hypertension, glomerulonephritis, and cystic kidney disease (Gupta et al., 2021). These observations highlight the profound cardiometabolic origins of kidney failure and the urgent need for therapies that improve cardiovascular outcomes in this high-risk population.

Renin–angiotensin system (RAS) inhibition with either angiotensin-converting enzyme inhibitors (ACEIs) or angiotensin receptor blockers (ARBs) is a foundational therapy for patients with hypertension and chronic kidney disease (CKD) (Natale et al., 2024; Hsu et al., 2014). Current hypertension and CKD guidelines generally consider ACEIs and ARBs as therapeutically interchangeable first-line RAS inhibitors, and direct head-to-head comparative evidence between the two classes has historically been limited (Jones et al., 2025). Nevertheless, emerging data suggest that subtle differences may exist in specific patient populations. For example, a large network meta-analysis of more than 40,000 patients with CKD stage 3–5 found that ACEIs were associated with superior renal and cardiovascular protection compared with other antihypertensive classes (Zhang et al., 2020). In contrast, several observational studies in general hypertensive cohorts have suggested that ARBs may offer comparable or even better tolerability and clinical outcomes than ACEIs, suggesting that the assumption of class equivalence may not uniformly apply across all high-risk clinical settings (Chen et al., 2021; Xie et al., 2025).

However, whether these benefits extend to patients with ESKD receiving dialysis remains uncertain. Importantly, the cardiovascular pathophysiology in ESKD differs substantially from that in earlier CKD stages, with a greater contribution from arrhythmia, hemodynamic instability, and vascular calcification (Jankowski et al., 2021). Accordingly, the risk–benefit profile of ARBs and ACEIs may diverge in dialysis patients due to unique intradialytic hemodynamics, fluctuations in blood pressure, and differences in drug clearance (Bucharles et al., 2019). Despite the central role of RAS blockade in CKD care, direct comparative evidence of ARBs versus ACEIs in patients with ESKD receiving maintenance dialysis remains sparse, and existing studies are limited by small sample sizes or lack of contemporary real-world representation (Shireman et al., 2016).

To address this knowledge gap, we conducted a large, multi-institutional, real-world cohort study to compare the 1-year cardiovascular outcomes associated with ARBs versus ACEIs therapy in patients with ESKD receiving maintenance hemodialysis. The primary endpoint was major adverse cardiovascular events (MACE), with secondary endpoints including myocardial infarction (AMI), stroke, and all-cause mortality. This study aimed to examine the comparative cardiovascular outcomes associated with different RAS inhibitors in patients receiving maintenance hemodialysis, a population in whom direct comparative evidence remains limited.

## Methods

### Data source

This retrospective cohort study was conducted using data from TriNetX, a large international federated network that compiles electronic health records from roughly 188 million patients across 164 healthcare organizations. The platform provides aggregated, de-identified data outputs on diagnoses, treatments, laboratory results, and genetic information without allowing access to protected health information or direct patient interaction. As a result, the study met criteria for exemption by the Western Institutional Review Board. All study procedures and reporting adhered to the guidelines outlined in the Strengthening the Reporting of Observational Studies in Epidemiology (STROBE) statement (Cuschieri, 2019).

### Study design

We identified individuals aged 18 years or older with a documented diagnosis of end-stage kidney disease (ESKD; ICD-10-CM Z99.2 and related procedural codes) who newly initiated therapy with either an ARB or an ACEI between 1 January 2006, and 30 September 2025. Patients with any prior or concurrent exposure to either drug before the index date were excluded. The index date was defined as the date of the first recorded prescription or administration of the assigned treatment. To reduce confounding by indication and align baseline risk, we implemented an active-comparator new-user design, comparing new initiators of ARBs with those initiating ACEIs. Individuals with evidence of study outcomes before follow-up were excluded to capture incident events only. We also removed patients who received the alternate drug within 1 year preceding or 1 year following the index date. To confirm sustained treatment exposure, patients were required to have a second prescription of the same medication between 1 day and 6 months after the index date. Detailed variable definitions and coding algorithms are provided in Supplementary Table S1.

### Covariates and propensity score matching

Following the identification of cohorts, index dates, study outcomes, and covariates, we compiled a baseline dataset using information recorded within the 12 months preceding the index date. Propensity scores were calculated using logistic regression to estimate each individual’s probability of initiating study drug based on baseline characteristics. Participants were subsequently matched in a 1:1 ratio using a greedy nearest-neighbor algorithm without replacement, applying a caliper of 0.1 of the pooled standard deviation of the logit of the propensity score. Covariate balance after matching was considered adequate when standardized mean differences were less than 0.1 (Haukoos and Lewis, 2015). Baseline covariates used for propensity score matching (PSM) were selected based on prior evidence to capture a broad range of demographic, clinical, and treatment-related factors that may influence therapy allocation and subsequent outcomes (Wu et al., 2025a; Wu et al., 2025b; Wu et al., 2025c; Wu et al., 2025d). Demographic variables included age (years, mean [SD]) and sex (female and male, n [%]), as well as race, categorized as White, Black or African American, and Asian (all n [%]).

Clinical comorbidities included heart failure, atrial fibrillation or flutter, nicotine dependence, chronic obstructive pulmonary disease, cirrhosis, and type 2 diabetes mellitus with neurological, kidney, circulatory, or ophthalmic complications (all n [%]). Medication exposure was characterized by the use of beta-blockers, calcium channel blockers, platelet aggregation inhibitors, and peripheral vasodilators (all n [%]). Physiological and laboratory parameters were evaluated using systolic blood pressure (mmHg, mean [SD] and ≥140 mmHg, n [%]), hemoglobin A1c (%, mean [SD] and ≥9%, n [%]), and low-density lipoprotein cholesterol (mg/dL, mean [SD] and ≥160 mg/dL, n [%]). Detailed coding definitions and measurement procedures for these covariates are provided in Supplementary Table S2.

### Outcomes and follow-up

The primary endpoint was a composite outcome of myocardial infarction, stroke, or all-cause mortality (hereafter referred to as MACE). All-cause mortality was included instead of cardiovascular mortality due to limitations in cause-of-death ascertainment within the database. Secondary endpoints included the individual occurrence of AMI, stroke, all-cause mortality, and hyperkalemia as a safety outcome. To assess potential residual confounding and evaluate the robustness of the findings, skin cancer was examined as a negative control outcome. Follow-up for outcomes began the day after the index date and continued until the earliest of the outcome event, last clinical encounter, death, or 1 year after cohort entry. Comprehensive definitions and coding algorithms for all outcomes are summarized in Supplementary Table S3.

### Subgroup analysis

For the primary outcome, prespecified subgroup analyses were performed by stratifying participants according to sex, age group (18–64 or ≥65 years), and key comorbid conditions. Comorbidity-based strata included diabetes mellitus (DM), heart failure, coronary artery disease (CAD), and peripheral artery disease (PAD).

### Statistical analysis

Continuous variables were described using means and standard deviations, while categorical variables were reported as counts and percentages. Baseline characteristics between treatment groups were balanced through propensity score matching before conducting the primary, subgroup, and sensitivity analyses. Hazard ratios (HRs) and 95% confidence intervals (CIs) were estimated using Cox proportional hazards regression. Time-to-event outcomes were further evaluated with Kaplan–Meier curves, and differences between groups were tested using the log-rank method. To evaluate the influence of potential unmeasured confounders, E-values were calculated. All statistical procedures were executed within the TriNetX analytics platform (VanderWeele and Ding, 2017).

## Results

### Study cohort

**FIGURE 1:**
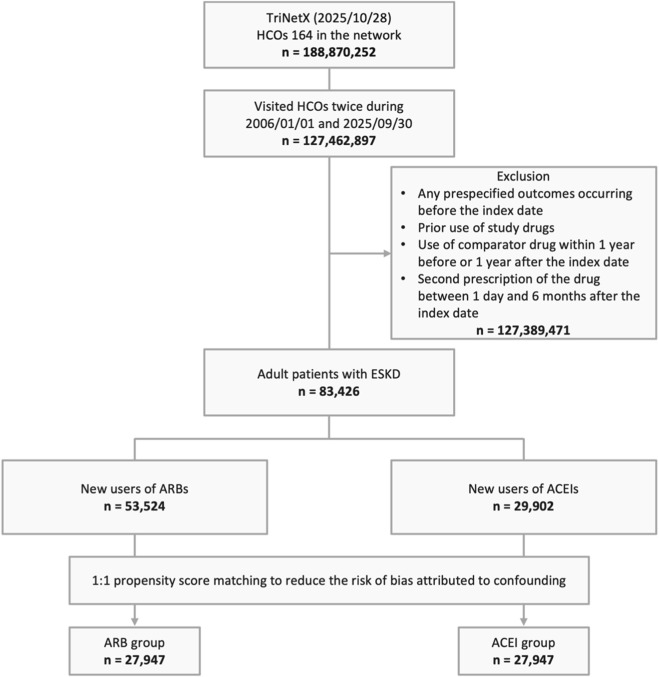
Study cohort process. ACEI, angiotensin-converting enzyme inhibitor; ARB, angiotensin II receptor antagonist; ESKD, end stage kidney disease; HCOs, healthcare organizations.

127,462,897 patients had at least two documented healthcare encounters between 1 January 2006, and 30 September 2025. After applying exclusion criteria, 83,426 adults with ESKD were eligible for inclusion. Of these, 53,524 patients were identified as new users of ARBs, and 29,902 as new users of ACEIs. After PSM, two balanced cohorts of 27,947 patients each in the ARB and ACEI groups (Figure 1).

### Characteristics of study subjects

Before matching, notable differences were observed between ARB and ACEI users in several demographic and clinical characteristics (Table 1). The ARB group was older on average compared with the ACEI group (61.4 ± 14.7 vs. 57.6 ± 16.4 years) and had lower proportions of White individuals and higher proportions of Asian individuals. Prevalence of comorbidities was generally higher among ACEI users, including heart failure (33.9% vs. 26.1%), atrial fibrillation or flutter (14.9% vs. 12.1%), and nicotine dependence (11.8% vs. 7.5%). Use of cardiovascular medications, such as beta-blockers and calcium channel blockers, was also more frequent in the ACEI group. Mean systolic blood pressure was slightly lower in ACEI users, whereas lipid and glycemic profiles were broadly similar between groups.

**TABLE 1:** Baseline characteristics of ARB and ACEI groups before and after matching.

Variables	Before matching			After matching		
	ARB group (n = 53,524)	ACEI group (n = 29,902)	Standardized difference	ARB group (n = 27,947)	ACEI group (n = 27,947)	Standardized difference
Age, years						
Mean (SD)	61.4 (14.7)	57.6 (16.4)	0.24	58.5 (15.3)	58.3 (16.1)	0.012
Sex, n (%)						
Female	22,487 (42)	11,514 (38.6)	0.069	11,133 (39.8)	11,147 (39.9)	0.001
Male	31,018 (58)	18,286 (61.4)	0.069	16,812 (60.2)	16,795 (60.1)	0.001
Race, n (%)						
White	13,711 (25.6)	13,418 (45)	0.414	12,009 (43)	12,008 (43)	0
Black or African American	11,000 (20.6)	8,545 (28.7)	0.189	8,314 (29.7)	8,287 (29.7)	0.002
Asian	23,144 (43.3)	3,140 (10.5)	0.794	3,140 (11.2)	3,140 (11.2)	0
Comorbidities, n (%)						
Heart failure	13,971 (26.1)	10,114 (33.9)	0.171	9,795 (35)	9,436 (33.8)	0.027
Atrial fibrillation and flutter	6,493 (12.1)	4,439 (14.9)	0.081	4,212 (15.1)	4,110 (14.7)	0.01
Nicotine dependence	4,003 (7.5)	3,525 (11.8)	0.148	3,219 (11.5)	3,167 (11.3)	0.006
Chronic obstructive pulmonary disease	3,865 (7.2)	2,565 (8.6)	0.051	2,442 (8.7)	2,394 (8.6)	0.006
Cirrhosis	1,861 (3.5)	1,045 (3.5)	0.002	1,020 (3.7)	978 (3.5)	0.008
Type 2 diabetes mellitus with neurological complications	4,617 (8.6)	3,136 (10.5)	0.064	2,989 (10.7)	3,047 (10.9)	0.007
Type 2 diabetes mellitus with kidney complications	14,584 (27.3)	8,988 (30.2)	0.064	9,064 (32.4)	8,877 (31.8)	0.014
Type 2 diabetes mellitus with circulatory complications	2,999 (5.6)	1,790 (6)	0.017	1,768 (6.3)	1,778 (6.4)	0.001
Type 2 diabetes mellitus with ophthalmic complications	3,240 (6.1)	2,181 (7.3)	0.051	2,108 (7.5)	2,098 (7.5)	0.001
Medications, n (%)						
Beta blockers	24,628 (46)	16,042 (53.8)	0.156	15,368 (55)	14,972 (53.6)	0.028
Calcium channel blockers	26,057 (48.7)	12,549 (42.1)	0.133	12,267 (43.9)	12,207 (43.7)	0.004
Platelet aggregation inhibitors	16,445 (30.7)	10,844 (36.4)	0.12	10,154 (36.3)	9,908 (35.5)	0.018
Peripheral vasodilators	1,573 (2.9)	807 (2.7)	0.014	722 (2.6)	702 (2.5)	0.005
Systolic blood pressure, mmHg						
Mean (SD)	143.2 (24.5)	139.6 (26.8)	0.141	143.4 (26.6)	140 (26.8)	0.127
≥140, n (%)	32,937 (61.6)	16,542 (55.5)	0.123	15,693 (56.2)	15,623 (55.9)	0.005
Hemoglobin A1c, %						
Mean (SD)	6.5 (1.7)	6.5 (1.9)	0.016	6.4 (1.7)	6.5 (1.9)	0.045
≥9, n (%)	2,461 (4.6)	1,331 (4.5)	0.006	1,254 (4.5)	1,267 (4.5)	0.002
Cholesterol in LDL, mg/dL						
Mean (SD)	88.5 (42.5)	80 (41.1)	0.204	81.8 (40.5)	80.6 (41.4)	0.029
≥160, n (%)	1,197 (2.2)	473 (1.6)	0.047	476 (1.7)	458 (1.6)	0.005

eGFR, estimated Glomerular filtration rate; LDL, low-density lipoprotein; SD, standard deviation.

After PSM, baseline characteristics were well balanced, with standardized differences below 0.1 for all variables (Table 1). Mean age, sex, and racial distribution were comparable between treatment groups. Comorbidities, including heart failure, atrial fibrillation, and diabetes-related complications, were evenly represented, as were concomitant cardiovascular medications. Physiologic and laboratory measures, including systolic blood pressure, hemoglobin A1c, and LDL cholesterol, showed minimal residual imbalance.

### Primary and secondary outcomes

During follow-up, 8,461 events of the primary composite endpoint occurred in the ARB group (30.3%) compared with 9,781 events in the ACEI group (35.0%), corresponding to a HR of 0.85 (95% CI, 0.83–0.88; p < 0.001; E-value, 1.6; Table 2).

**TABLE 2:** Hazard ratio of outcomes between ARB and ACEI groups.

Outcome	ARB group (n = 27,947)	ACEI group (n = 27,947)	HR (95% CI)	*P* value	E-value (95% LCL)
	Events (%)	Events (%)			
Primary outcome					
MACE	8,461 (30.3)	9,781 (35.0)	0.85 (0.83,0.88)	<0.001	1.6 (1.5)
Secondary outcomes					
AMI	3,279 (11.7)	3,357 (12.0)	0.99 (0.94,1.03)	0.527	1.1 (1.0)
Stroke	4,052 (14.5)	4,513 (16.1)	0.90 (0.86,0.94)	<0.001	1.5 (1.3)
All-cause mortality	3,034 (10.9)	4,085 (14.6)	0.75 (0.71,0.78)	<0.001	2.0 (1.9)
Hyperkalemia	2,767 (9.9)	2,964 (10.6)	1.00 (0.95,1.05)	0.849	1.0 (1.0)

ACEI, angiotensin-converting enzyme inhibitor; AMI, acute myocardial infarction; ARB, angiotensin II, receptor antagonist; CI, confidence interval; HR, hazard ratio; LCL, lower confidence limit; MACE, major adverse cardiovascular event.

**FIGURE 2:**
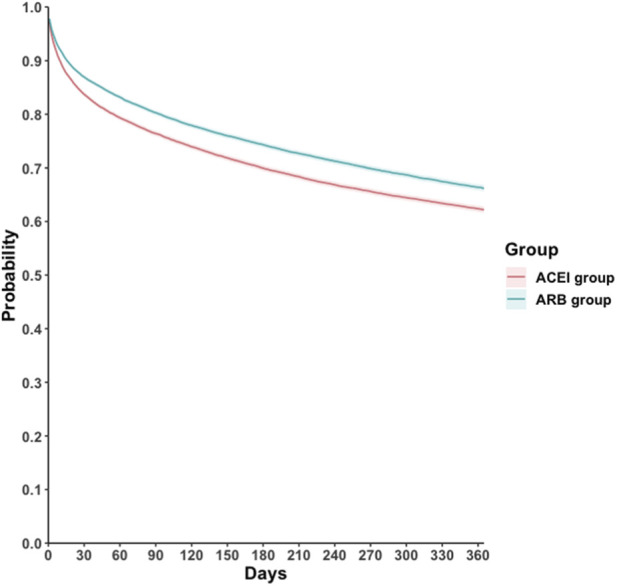
Kaplan-Meier time-to-event free curves of primary outcome comparison to ARB and ACEI groups. ACEI, angiotensin-converting enzyme inhibitor; ARB, angiotensin II receptor antagonist.

Kaplan-Meier curves demonstrated a consistently higher primary outcome-free probability in the ARB group than in the ACEI group (log-rank P < 0.001, Figure 2).

For secondary outcomes, rates of AMI were similar between groups (11.7% vs. 12.0%), with no significant difference in risk (HR, 0.99; 95% CI, 0.94–1.03; p = 0.527; Table 2). Stroke occurred in 4,052 patients in the ARB group (14.5%) and 4,513 patients in the ACEI group (16.1%), yielding an HR of 0.90 (95% CI, 0.86–0.94; p < 0.001; E-value, 1.5; Table 2). All-cause mortality was observed less frequently in ARB users (10.9% vs. 14.6%), with an HR of 0.75 (95% CI, 0.71–0.78; p < 0.001; E-value, 2.0; Table 2). Hyperkalemia occurred in 9.9% and 10.6% of patients, respectively, with no statistically significant difference between groups (HR, 1.00; 95% CI, 0.95–1.05; p = 0.849; Table 2).

### Subgroup analysis

**FIGURE 3:**
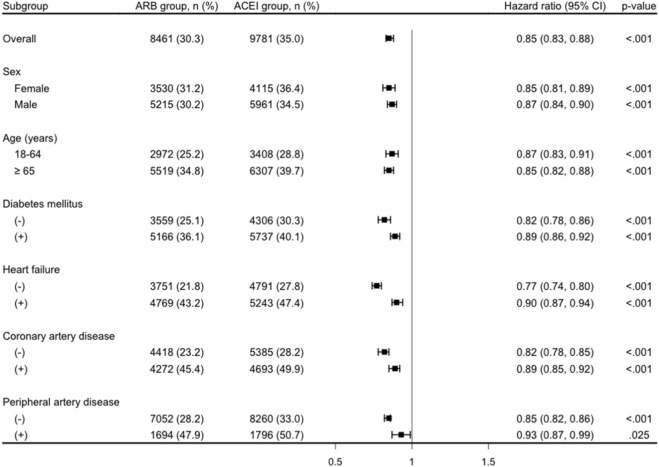
Subgroup analysis for the risk of primary outcome comparison to ARB and ACEI groups. ACEI, angiotensin-converting enzyme inhibitor; ARB, angiotensin II receptor antagonist; CI, confidence interval.

In subgroup analyses of the primary outcome, HRs consistently favored ARB over ACEI therapy across all clinical strata (Figure 3). By sex, the HR was 0.85 (95% CI, 0.81–0.89; p < 0.001) among females and 0.87 (95% CI, 0.84–0.90; p < 0.001) among males. Across age groups, HRs were 0.87 (95% CI, 0.83–0.91; p < 0.001) for individuals aged 18–64 years and 0.85 (95% CI, 0.82–0.88; p < 0.001) for those aged ≥65 years. When stratified by DM status, HRs were 0.82 (95% CI, 0.78–0.86; p < 0.001) for individuals without DM and 0.89 (95% CI, 0.86–0.92; p < 0.001) for those with DM. Among those without heart failure, the HR was 0.77 (95% CI, 0.74–0.80; p < 0.001), whereas in those with heart failure, the HR was 0.90 (95% CI, 0.87–0.94; p < 0.001). For CAD, HRs were 0.82 (95% CI, 0.78–0.85; p < 0.001) in individuals without the condition and 0.89 (95% CI, 0.85–0.92; p < 0.001) in those with it. In patients without PAD, the HR was 0.85 (95% CI, 0.82–0.86; p < 0.001), while among those with the condition, the HR was 0.93 (95% CI, 0.87–0.99; p = 0.025).

## Discussion

In this large, multi-institutional, real-world cohort of 55,894 patients with ESKD receiving maintenance hemodialysis, we observed differences in 1-year cardiovascular outcomes between patients initiating ARBs and those initiating ACEIs. This benefit was primarily driven by all-cause mortality and stroke, whereas the risks of AMI was comparable between groups. Subgroup and sensitivity analyses were consistent with the primary findings, and safety outcomes were similar across treatment groups. The negative control outcome analysis yielded null associations, supporting the robustness of our results and minimizing concerns about unmeasured confounding or coding-related bias. Notably, the magnitude of mortality reduction observed in this study is substantial and may not be fully explained by measured confounders, raising the possibility of residual confounding or selection bias. Furthermore, the magnitude of reduction in all-cause mortality observed in this study is substantial and should be interpreted with caution. Given the observational design, residual confounding, particularly related to patient selection, treatment tolerance, and unmeasured clinical factors, may have contributed to the apparent effect size.

An important finding of this study is that the observed reduction in the composite outcome was primarily driven by stroke and all-cause mortality, whereas the risk of myocardial infarction was similar between groups. This pattern may reflect differences in the underlying pathophysiology of cardiovascular disease in patients receiving maintenance hemodialysis.

In this population, cardiovascular mortality is frequently driven by non-atherosclerotic mechanisms, including arrhythmia, sudden cardiac death, intradialytic hemodynamic instability, and structural heart disease, rather than acute coronary plaque rupture (Jankowski et al., 2021; Aoki and Ikari, 2017). In contrast, myocardial infarction represents a more traditional atherosclerotic endpoint, which may be less sensitive to differences between RAS inhibitor classes in this setting. Additionally, stroke risk in dialysis patients is influenced by blood pressure variability and vascular stiffness, which may be differentially affected by RAS inhibition and could partially explain the observed association (Bucharles et al., 2019).

Guidelines for hypertension and CKD care traditionally regard ACEIs and ARBs as therapeutically interchangeable RAS inhibitors, and direct comparative evidence has been limited (Jones et al., 2025; Kidney Disease: Improving Global Outcomes CKD Work Group, 2024). In a large multinational cohort study of 2,297,881 patients with hypertension, no statistically significant differences were observed between ACEIs and ARBs in the risks of AMI (HR 1.11; 95% CI 0.95–1.32), heart failure (HR 1.03; 95% CI 0.87–1.24), stroke (HR 1.07; 95% CI 0.91–1.27), or composite MACE (HR 1.06; 95% CI 0.90–1.25) (Chen et al., 2021). Notably, however, ARBs were associated with a substantially lower risk of adverse effects compared with ACEIs, suggesting a more favorable tolerability profile. In contrast, another systematic review and meta-analysis including 13 randomized trials and 47,008 patients with hypertension and diabetes reported that ACEIs therapy was associated with significant reductions in several cardiovascular endpoints, such as cardiovascular death (OR 0.81; 95% CI 0.68–0.98), AMI (OR 0.77; 95% CI 0.66–0.90), stroke (OR 0.88; 95% CI 0.78–0.99), and heart failure (OR 0.65; 95% CI 0.47–0.90), compared with placebo (Lv et al., 2018). In contrast, ARB therapy did not demonstrate significant reductions across many primary or secondary cardiovascular outcomes in the included trials (Lv et al., 2018). In another network meta-analysis of 44 randomized clinical trials including 42,319 patients with non-dialysis CKD, ACEIs remained superior to ARBs and other antihypertensive agents, providing the greatest benefits in reducing kidney events, cardiovascular outcomes, cardiovascular death, and all-cause mortality (Zhang et al., 2020). However, these advantages came at the cost of higher rates of hyperkalemia, cough, and hypotension (Zhang et al., 2020). Taken together, these findings suggest that ACEIs and ARBs may exert differential benefits across distinct patient populations, rather than functioning as fully interchangeable agents in all clinical settings.

Evidence directly comparing ARBs and ACEIs in the dialysis population remains limited. A prior national retrospective cohort study including 4,997 hemodialysis patients reported that ACEIs users had higher risks of all-cause mortality (adjusted HR 1.22; 99% CI 1.05–1.42) and cardiovascular events (adjusted HR 1.12; 99% CI 0.99–1.27) compared with ARBs users (Shireman et al., 2016). However, that study was based on a relatively small sample size and reflected earlier-era dialysis practice patterns, limiting the generalizability to contemporary populations. Consequently, whether ARBs and ACEIs confer differential prognostic effects in patients receiving maintenance dialysis has remained uncertain. Our study helps fill this important evidence gap. Using a large, contemporary, multi-institutional real-world cohort, we observed that initiation of ARBs was associated with lower risks of major adverse cardiovascular events, stroke, and all-cause mortality compared with ACEIs in patients with ESKD receiving maintenance hemodialysis. These findings are consistent with prior observational reports suggesting an association between ARB use and lower mortality, which may extend to contemporary dialysis populations.

The cardiovascular disease mechanisms in dialysis-dependent ESKD differ markedly from those in earlier CKD stages (Aoki and Ikari, 2017). Sudden cardiac death, left ventricular hypertrophy, vascular calcification, and hemodynamic instability account for a disproportionate share of mortality in hemodialysis patients, whereas classic atherosclerotic events such as AMI play a comparatively smaller role (Charytan, 2014). These mechanisms may partially account for the observed associations between ARB use and lower mortality. First, ACEIs increase bradykinin levels, which can exacerbate intradialytic hypotension, a common and clinically important complication associated with arrhythmia, cardiac stunning, and reduced perfusion to vital organs (Gamboa et al., 2023; Campbell, , 1995; Kanbay et al., 2020). ARBs do not affect bradykinin pathways and may therefore be associated with more stable hemodynamic profiles during dialysis. Second, ARBs have been suggested to exert effects on vascular remodeling and left ventricular hypertrophy regression in prior studies, which are key determinants of mortality in ESKD (Yang et al., 2013; Chen et al., 2020). Third, inflammation and vascular calcification, both prominent in dialysis patients, may be differentially modulated by ARBs through more selective AT1 receptor blockade (MacKenzie, 2011; Xia et al., 2018; Moe and Chen, 2005). Taken together, these mechanisms may provide plausible explanations for the observed associations, although causality cannot be established. From a clinical perspective, these findings suggest that the assumption of equivalence between ACEIs and ARBs may not fully capture observed differences in certain high-risk populations; however, these hypotheses cannot be confirmed within the scope of this study. Given the unique cardiovascular risk profile and hemodynamic vulnerability of this population, further prospective and pragmatic studies are warranted to clarify optimal RAS inhibitor selection (Cozzolino et al., 2018).

### Limitations

This study has several limitations. First, the TriNetX platform is a registry-based database, and the potential for patient misclassification or underrepresentation, particularly among individuals with milder disease severity or limited healthcare engagement, may affect the generalizability of our findings. In addition, the reliance on diagnostic and procedural coding to ascertain exposures, covariates, and outcomes introduces the possibility of misclassification bias. Furthermore, detailed clinical data, including dialysis adequacy, blood pressure variability, and cause-specific mortality, were not available, which may limit mechanistic interpretation. To mitigate this concern, we incorporated negative control outcome analyses using clinically unrelated conditions, which showed no significant differences between groups and therefore suggest minimal coding-related bias. Additionally, although a new-user design and exposure definition were applied, the possibility of immortal time bias cannot be fully excluded. Second, the TriNetX platform does not provide detailed information on medication dose, treatment adherence, or longitudinal treatment changes, precluding assessment of dose–response relationships and time-varying exposure effects. Medication exposure was defined based on prescription records and may not fully reflect actual drug adherence or persistence. In addition, Cox proportional hazards models were not stratified by matched pairs due to platform limitations, which may lead to conservative variance estimation. Furthermore, competing risks, particularly death, were not formally accounted for in analyses of nonfatal outcomes such as stroke and myocardial infarction, as Fine–Gray models are not supported within the platform. These methodological constraints should be considered when interpreting the results.

Third, although propensity score matching was applied to balance baseline characteristics, several clinically relevant factors were not captured in the database and may contribute to residual confounding. These include patient frailty, dialysis adequacy, blood pressure variability, and medication adherence, all of which may influence both treatment selection and clinical outcomes.

Furthermore, indication bias may be present. For example, ARBs may be preferentially prescribed in patients who are intolerant to ACEIs, such as those with cough or hypotension. These patients may differ systematically from ACEI users in ways not fully captured by measured covariates. The direction of bias introduced by these factors is difficult to determine with certainty. However, it is possible that unmeasured confounding, particularly related to patient frailty or treatment selection, may have influenced the observed associations and potentially contributed to the magnitude of the apparent benefit. We therefore calculated E-values, which indicated only moderate robustness to unmeasured confounding. Therefore, residual confounding of moderate magnitude could still influence the observed associations, and the findings should be interpreted with caution. Importantly, E-value analysis does not account for multiple unmeasured confounders acting jointly and should be interpreted as a sensitivity analysis rather than definitive evidence of causal robustness. Fourth, although we required a second prescription within 1 day–6 months to enhance exposure validity, this design choice may introduce selection bias by preferentially including patients who survived long enough and remained adherent to receive continued treatment. This could result in a cohort that is healthier or more clinically stable than the broader population of treatment initiators. Although follow-up time was defined from the index date to mitigate classical immortal time bias, the possibility of residual selection bias cannot be fully excluded. The direction of this bias may favor patients with better adherence and overall prognosis, potentially leading to an overestimation of treatment benefit. Fifth, the composite endpoint of MACE included all-cause mortality rather than cardiovascular mortality, which differs from conventional definitions used in many cardiovascular trials. This approach was adopted because cause-specific mortality is not reliably captured in the TriNetX platform, and restricting analyses to cardiovascular death may introduce misclassification bias. In addition, in patients with end-stage kidney disease receiving hemodialysis, all-cause mortality represents a clinically meaningful endpoint due to the high competing risk of non-cardiovascular death and the difficulty in accurately attributing cause of death. However, this definition may limit direct comparability with prior studies using traditional MACE definitions, and the findings should be interpreted in this context.

Finally, cause-specific mortality data were not accessible, preventing us from distinguishing cardiovascular from non-cardiovascular deaths and potentially leading to endpoint misclassification. In addition, the relatively short follow-up duration of 1 year limits the ability to assess long-term cardiovascular outcomes and may not fully capture the cumulative effects of RAS inhibition over time.

## Conclusion

In this large, multi-institutional real-world cohort of patients with ESKD receiving maintenance hemodialysis, ARB therapy was associated with lower risks of MACE, stroke, and all-cause mortality compared with ACEIs. However, given the observational design, these findings should be interpreted with caution and should be considered hypothesis-generating and require confirmation in prospective studies.

## Data Availability

The data analyzed in this study is subject to the following licenses/restrictions: The datasets used in this study are derived from the TriNetX research network and are not publicly available. Access is restricted to authorized users through a subscription-based platform. The data consist of de-identified electronic health records, and individual-level data cannot be downloaded or shared in order to protect patient privacy and comply with data use agreements. Requests to access these datasets should be directed to https://trinetx.com/.
