# Supplementary material for: Comparative cardiovascular outcomes of renin–angiotensin system inhibitors in patients receiving maintenance hemodialysis: a large real-world cohort study
Source: Front Pharmacol. 2026 Jun 15;17:1833065. doi: 10.3389/fphar.2026.1833065 (PMC13312885; doi:10.3389/fphar.2026.1833065)
Supplement: Supplementary file 1 [file Table1.docx]

**Table S1.** Demographic, diagnostic, procedural, medication, visit, and laboratory codes utilized in the definition of the cohorts.

| **Category** | **Code** | **Description** |
| --- | --- | --- |
| **Index drug** | | |
| medication | NLM:VA:CV805 | ANGIOTENSIN II INHIBITOR |
| medication | NLM:VA:CV800 | ACE INHIBITORS |
| **ESKD** | | |
| diagnosis | UMLS:ICD10CM:Z99.2 | Dependence on renal dialysis |
| procedure | UMLS:CPT:1012740 | Dialysis Services and Procedures |
| procedure | UMLS:CPT:90937 | Hemodialysis procedure requiring repeated evaluation(s) with or without substantial revision of dialysis prescription |
| procedure | UMLS:CPT:90945 | Dialysis procedure other than hemodialysis (eg, peritoneal dialysis, hemofiltration, or other continuous renal replacement therapies), with single evaluation by a physician or other qualified health care professional |
| procedure | UMLS:CPT:90947 | Dialysis procedure other than hemodialysis (eg, peritoneal dialysis, hemofiltration, or other continuous renal replacement therapies) requiring repeated evaluations by a physician or other qualified health care professional, with or without substantial revision of dialysis prescription |
| procedure | UMLS:CPT:90935 | Hemodialysis procedure with single evaluation by a physician or other qualified health care professional |
| procedure | UMLS:SNOMED:302497006 | Hemodialysis |
| procedure | UMLS:CPT:1012752 | Hemodialysis Procedures |

**Table S2.** Definitions of covariates coding used in this study.

| **Code** | **Description** |
| --- | --- |
| AI | Age at Index |
| 2106-3 | White |
| F | Female |
| 2054-5 | Black or African American |
| M | Male |
| 2028-9 | Asian |
| F17 | Nicotine dependence |
| I50 | Heart failure |
| I48 | Atrial fibrillation and flutter |
| E11.2 | Type 2 diabetes mellitus with kidney complications |
| E11.3 | Type 2 diabetes mellitus with ophthalmic complications |
| E11.4 | Type 2 diabetes mellitus with neurological complications |
| E11.5 | Type 2 diabetes mellitus with circulatory complications |
| J44.9 | Chronic obstructive pulmonary disease, unspecified |
| K74 | Fibrosis and cirrhosis of liver |
| C04 | PERIPHERAL VASODILATORS |
| C08 | CALCIUM CHANNEL BLOCKERS |
| C07 | BETA BLOCKING AGENTS |
| BL117 | PLATELET AGGREGATION INHIBITORS |
| 9037 | Hemoglobin A1c/Hemoglobin.total in Blood |
| 9002 | Cholesterol in LDL [Mass/volume] in Serum or Plasma |
| 9085 | Blood Pressure, Systolic |

**Table S3.** Definitions of outcomes coding used in this study.

| **Code** | **Description** |
| --- | --- |
| **AMI** | |
| UMLS:ICD10CM:I21 | Acute myocardial infarction |
| UMLS:ICD10CM:I22 | Subsequent ST elevation (STEMI) and non-ST elevation (NSTEMI) myocardial infarction |
| **MACE** | |
| Deceased | Deceased |
| UMLS:ICD10CM:R99 | Ill-defined and unknown cause of mortality |
| UMLS:ICD10CM:I21 | Acute myocardial infarction |
| UMLS:ICD10CM:I63 | Cerebral infarction |
| NLM:RXNORM:8410 | alteplase |
| UMLS:ICD10CM:I22 | Subsequent ST elevation (STEMI) and non-ST elevation (NSTEMI) myocardial infarction |
| **Stroke** | |
| UMLS:ICD10CM:I63 | Cerebral infarction |
| NLM:RXNORM:8410 | alteplase |
| **Hyperkalemia** | |
| UMLS:ICD10CM:E87.5 | Hyperkalemia |
| **All-cause mortality** | |
| Deceased | Deceased |
| UMLS:ICD10CM:R99 | Ill-defined and unknown cause of mortality |

**Table S4**. Negative outcome control.

| Outcome | HR (95% CI) | *P* value |
| --- | --- | --- |
|  |  |  |
| Skin cancer | 1.21 (0.90,1.64) | 0.205 |
